# Implication of miR-122, miR-483, and miR-335 Expression Levels as Potential Signatures in HCV-Related Hepatocellular Carcinoma (HCC) in Egyptian Patients

**DOI:** 10.3389/fmolb.2022.864839

**Published:** 2022-05-16

**Authors:** Ashraf Y. Elfert, Amel Salem, Amr M. Abdelhamid, Ahmad Salama, Doaa A. Sourour, Olfat Shaker, Mofida Keshk

**Affiliations:** ^1^ Clinical Biochemistry and Molecular Diagnostics, National Liver Institute, Menoufia University, Menoufia, Egypt; ^2^ Department of Internal Medicine, Faculty of Medicine, Cairo University, Giza, Egypt; ^3^ Biochemistry Department, Faculty of Pharmacy, October University for Modern Sciences and Arts (MSA), 6th of October, Egypt; ^4^ Tropical Medicine Department, Faculty of Medicine, Cairo University, Cairo, Egypt; ^5^ Department of Medical Research and Radiation, Nuclear Materials Authority, Cairo, Egypt; ^6^ Department of Medical Biochemistry and Molecular Biology, Faculty of Medicine, Cairo University, Giza, Egypt; ^7^ Department of Molecular Diagnostics and Therapeutics, Genetic Engineering and Biotechnology Research Institute, University of Sadat City, Cairo, Egypt

**Keywords:** HCV, HCC, miRNA-122, miRNA-483, miRNA-335

## Abstract

Hepatocellular carcinoma (HCC) is the second most common cause of cancer-related deaths worldwide with chronic hepatitis C virus (HCV) infection as a major risk factor of HCC. Circulating microRNAs are deregulated in HCC and are candidate biomarkers. The aim of this study was to explore the expression profile of miRNA-122, miR-483, and miR-335 in the serum of HCV-related hepatocellular carcinoma (HCC). 90 HCV-related hepatocellular carcinoma (HCC) patients, 90 non-malignant HCV patients, and 60 healthy controls were included. Serum microRNAs were measured by a qRT-PCR custom array. The expression levels of miR-122 and miR-483 were upregulated in HCC patients, while the miR-335 expression level was downregulated versus controls and HCV groups. Receiver-operating characteristic (ROC) curve analysis was created to examine miRNAs. miR-483 presented the best diagnostic potential because it showed the highest diagnostic accuracy for distinguishing HCV-related HCC patients from controls (AUC = 0.98) with 100% sensitivity. Moreover, there was obvious prognostic power in distinguishing HCV from HCC (AUC = 0.95) with 88% sensitivity. In conclusion, studied microRNAs (miR-122, miR-483, and miR-335) could serve as potential non-invasive early diagnostic biomarkers for HCC, and we identified a panel of three serum microRNAs with high accuracy in HCC diagnosis. Additional studies are required to confirm this panel and test its prognostic significance.

## Introduction

Hepatocellular carcinoma (HCC) is the world’s second-leading cause of cancer-related deaths (De Toni et al., 2020), with chronic hepatitis C virus (HCV) infection being a key risk factor (Ozakyol, 2017). It is worth noting that HCC accounts for 13% of all malignancies in Egypt and is the second most common disease in men. In Egypt in 2010, chronic HCV accounted for 94 percent of HCC cases, with 6,000–7,000 fatalities per year owing to HCC (Fathy.Elmougy et al., 2019). Since they are protected from RNases, circulating miRNAs have been proven to have a high level of stability (Cui et al., 2019) even under extreme conditions like boiling, long storage, and many freeze–thaw cycles, with extremely low or high pH (Drees and Pegtel, 2020). These findings suggest that miRNAs are widely distributed and may be effective in the early detection of HCC (Shen et al., 2013).

MiRNA-122 (miR-122) expression is restricted to the liver, where it is estimated to account for 70% of all miRNA expression. miR-122 has been shown to have a role in hepatocarcinogenesis, and variations in the circulating amount of miR-122 were detected in patients with HCC compared to healthy people (Zhao et al., 2020). In addition, miR-122 was found to have a vital role in the infection of hepatitis C virus (Panigrahi et al., 2021). Deregulation of miR-122 has been linked to an aggressive kind of HCC in several investigations (Franck et al., 2020). miR-122 was first identified in HCC patients as a tumor suppressor gene (Fong et al., 2015). Also, miR-122 was detected in breast cancer patients as a tumor suppressor that targets the receptor of insulin-like growth factor-1 (IGF1R) (Wang et al., 2012). Other studies have revealed that miR-122 is a tumor suppressor through modulating oncogenes including CDK4, cyclin G1, and AKT3 in hepatocellular carcinoma (HCC) (Nassirpour et al., 2013). Meanwhile, another study demonstrated the overexpression of miR-122 in types of renal carcinoma (ccRCC) (Chow et al., 2010), implying miR-122 as an onco-microRNA in renal cancer (Fong et al., 2015). miR-122 physiological function in cancer tends to vary depending on the kind of cancer, and the exact mechanism through which miR-122 affects ccRCC growth is yet undetermined (Jingushi et al., 2017).

miR-483 was shown to be highly elevated in the serum of people who had HCV infection (Shwetha et al., 2013), suggesting that upregulation of miR-483-5 could be a mechanism for regulating various pathways involved in HCV pathogenesis or infection defense (Shwetha et al., 2013). In HCC cells, miR-483 promotes tumor invasion using activated leukocyte cell adhesion molecule (ALCAM) as a functional target (Tang et al., 2017). ALCAM is suggested to have a crucial role in proliferation and migration of cancer cells as a member of the immunoglobulin super family (Jiang et al., 2020). ALCAM expression and function varied among tumors; in certain tumors, it promotes malignancy, while in others, it suppresses it (Shen et al., 2013). These data suggest that the pathway of miR-483-5/ALCAM is a key regulator in HCC intrahepatic metastasis and that it can be used as a prognostic indicator and the base for tailored treatment (Lu et al., 2018). In cancer cell lines, miR-483-3p was discovered to modulate antiapoptotic oncogenes including HCT116 in colorectal carcinoma and HEPG2 in liver carcinoma. In addition, miR-483-5 was suggested to have a growth-promoting effect in an *in vitro* study of adrenocortical cancer. These findings suggest that miR-483-5 may have a malignant effect in carcinogenesis (Shen et al., 2013).

miR-335 promotes cancer growth by acting as an oncogene (Fouda et al., 2021). In gallbladder cancer, miR-335 suppresses the myocyte enhancer factor-2D (MEF2D) and improves cell proliferation as well as increasing the sensitivity of cancer cells to 5-fluorouracil chemotherapy (Ye et al., 2021). MEF2D is a member of the MEF2 family of transcription factors. In muscle, heart, and cancer cells, it can control cell division, differentiation, and apoptosis (McKinsey et al., 2002). Another study has demonstrated that miR-335 can promote cell proliferation and progression through targeting the cancer suppressor RAS-p21 protein activator-1 (RASA1) in CRC (Lu et al., 2016). The expression of miR-335 is aberrant in a range of malignancies (Zarfeshani et al., 2015); yet, it is still debated in some cancer types. Furthermore, the role of miR-335 in numerous cellular processes as well as the miR-335 complex regulatory networks is unknown.

The current study aimed to investigate the expression profiles of miR-122, miR-483, and miR-335 in sera from Egyptian patients with non-malignant HCV and HCV-related HCC in an attempt to use them as potential non-invasive biomarkers for HCV-related HCC diagnosis.

## Materials and Methods

### Subjects

A total of 60 healthy volunteers have participated in the current study (control group). They have normal liver function tests, normal alpha-fetoprotein (AFP) levels, normal hepatic ultrasonography, and negative results for HCV and HBV. In addition, the following HCV-infected patients were incorporated in the current study: 90 HCV-related HCC patients and 90 non-malignant HCV patients. They were hospitalized to the liver unit’s outpatient clinic of Kasr El-Aini Hospital (Cairo University). All of them tested positive for anti-HCV antibodies and had detectable HCV RNA in their blood. The diagnosis of HCC according to the European Association for the Study of the Liver criteria (2012) depended mainly on the presence of hepatic focal lesions discovered by abdominal ultrasonography and verified by magnetic resonance imaging or computed tomography. The Child–Pugh scale is used for grading the severity of hepatic disease in HCC, while the Barcelona Clinic Liver Cancer (BCLC) staging approach is used for HCC staging [15]. For each patient, a history was taken, a clinical examination was performed, and routine laboratory tests were performed. Patients with chronic HBV or any other detectable cause of chronic hepatitis besides HCV, with any preceded malignancies other than HCC, or who had previously had HCC or antiviral therapy for HCV were excluded from the study. All patients and controls gave their written informed consent for gene analysis. The study protocol that followed the Helsinki Declaration’s ethical principles and the informed consent were approved by the Ethics Committee of Faculty of Medicine (Cairo University, Egypt).

## Methods

### Laboratory Tests

All patients had fasting venous blood samples drawn for routine testing, which included a complete blood count, liver function tests, prothrombin concentration and international normalized ratio, AFP, and anti-HCV titer. All of these were investigated using available assays.

### Extraction and Reverse Transcription of RNA

Extraction of total RNA (including miRNAs) was performed using the miRNeasy extraction kit according to the manufacturer’s instructions (Qiagen, Valencia, CA). NanoDrop2000 was used to assess the quality of RNA (Thermo Scientific, United States). According to the producer’s instructions using the miScript II RT Kit (Qiagen, Valencia, CA), reverse transcription (RT) was performed on 100 ng of total RNA in a final volume of 20 µl RT reactions (incubated for 60 min at 37°C and 5 min at 95°C).

### Quantitative Real-Time PCR

The expression levels of miR-122, miR-483, and miR-335 in sera were measured using the miScript miRNA PCR custom array (Qiagen, Valencia, CA). As previously reported, as an internal control, the housekeeping miScript PCR control, miRNA SNORD68, was used (El- Garem et al., 2014). SNORD68 appears to be a reliable normalization control that could be used in miRNA PCR analysis, based on our past experience (Motawi et al., 2015). For real-time PCR of each miRNA, 2.5 μl diluted RT products were combined with 5.5 μl RNase-free water, 10 μl QuantiTect SYBR Green PCR Master Mix, and 2 μl miScript universal primer (reverse primer) and then added to a custom Rotor-Disc 100 miRNA PCR array that contains miRNA-specific miScript primer assays (Qiagen, Valencia, CA). Optical thin wall strips were used to seal Rotor-Disc. The Rotor-Gene Q real-time PCR system (Qiagen, Valencia, CA) was used to run real-time PCR under the following settings: 95°C for 30 min, followed by 40 cycles of 15 s at 94°C, 30 s at 55°C, and 30 s at 70°C. The cycle threshold (Ct) is the number of cycles required for the fluorescent signal to cross the threshold in real-time PCR. The fold change of miRNA expression levels was calculated using the 2-ΔΔCt formula (healthy controls were used as a calibrator): ΔΔCt = [Ct (target, test)-Ct (reference, test)]-[Ct (target, calibrator)-Ct (reference, calibrator)] (Livak and Schmittgen, 2001).

### Statistical Analysis

GraphPad prism software package version six was used to examine the data. The following statistical tests were used: the chi-square test and Kruskal–Wallis test to compare categorical variables between groups; the F-test (ANOVA) to compare normally distributed quantitative variables between more than two groups; and the post hoc test (Tukey) for pairwise comparisons. Plotting sensitivity (TP) on the *Y*-axis versus 1-specificity (FP) on the *X*-axis at various cut-off settings was used for generating the receiver-operating characteristic (ROC) curve. The diagnostic performance of a test is measured by the area under the ROC curve. The area more than 50% gives acceptable performance, and the area about 100% is the best performance for the test.

## Results

Gender did not differ substantially between the tested groups (*p* = 0.127). However, there was a male predominance of 80 percent, 73.3 percent, and 58.3 percent in HCC-related HCV, non-malignant HCV, and control groups, respectively. In addition, there was no significant difference between the studied groups in age (Table 1). Serum levels of AST, ALT, ALP, and GGT were significantly higher than the normal ranges in the HCC group. Albumin and prothrombin concentrations decreased significantly through the progression of liver disease among the HCC group (Table 2).

**TABLE 1:** Comparison between the studied groups according to demographic data.

Demographic data	HCC (*n* = 90)		HCV (*n* = 90)		Controls (*n* = 60)		Test of sig.	*p*
	No.	%	No.	%	No.	%		
Gender								
Male	72	80.0	66	73.3	35	58.3	*χ* ^2^ = 4.127	0.127
Female	18	20.0	24	26.7	25	41.7		
Age (years)								
							F = 1.727	0.184
Mean ± SD	47.43 ± 7.56		43.40 ± 11.66		44.0 ± 7.32			
Median (IQR)	47.50 (42.0–53.0)		47.0 (33.0–52.0)		42.0 (37.0–49.0)			

χ^2^, chi-square test.

F, F for ANOVA test.

*p*, *p* value for comparison between the different groups.

**TABLE 2:** Descriptive analysis of the studied cases according to different laboratory parameters for the HCC group (*n* = 90).

Laboratory parameters	Mean ± SD	Median (IQR)
TLC (x10^3^)	5.91 ± 2.28	5.75 (4.6–6.60)
Platelet count (ul/blood)	123.87 ± 75.74	110.0 (77.0–161.0)
AST (U/L)	124.83 ± 64.02	126.0 (67.0–167.0)
ALT ((U/L)	82.70 ± 49.93	70.50 (42.0–107.0)
ALP (U/L)	184.50 ± 161.81	160.0 (135.0–190.0)
GGT (U/L)	66.87 ± 56.43	55.0 (40.0–70.0)
Albumin	3.89 ± 1.38	3.50 (3.10–4.0)
Urea	11.92 ± 19.79	7.10 (6.70–8.0)
Creatinine	1.25 ± 0.75	1.0 (0.99–1.20)
Prothrombin time	16.06 ± 3.16	15.25 (14.2–16.10)
PCV	0.70 ± 0.15	0.73 (0.65–0.80)

Table 3 shows the clinicopathological features of HCC patients. At the time of diagnosis, HCC patients had a wide range of AFP values (mean SD), with normal AFP levels (up to 10 ng/ml) present in as many as 23.3 percent of patients, 35.5 percent having AFP >20 ng/ml, and 41.2 percent having AFP>400 ng/ml. HCC patients had Child–Pugh grade B, and 20% and 80% had stage C with the mean Child–Pugh score of 6.46 ± 1.36. 66.7% of the HCC patients had mild ascites, while 6.7% had severe ascites. In BCLC staging, 46.7% had grade B, 13.3% grade D, and 26.7% had grade C. The HCC patients had a mean “Model for End-Stage Liver Disease” (MELD) score of 8.40 ± 7.89. In patients with cirrhosis, alcoholic hepatitis, acute liver failure, and acute hepatitis, the MELD score has been proven as a predictor of survival. The number of extra-hepatic organ failures is more predictive of mortality in terminally sick cirrhotic patients than the MELD score, which ranges from 6 to 40.

**TABLE 3:** Distribution of the studied cases according to different parameters for the HCC group (*n* = 90).

	No.	%
AFP level		
<20 ng/ml	21	23.3
20–350 ng/ml	32	35.5
350 ng/ml	37	41.2
Child–Pugh score		
B	18	20.0
C	72	80.0
Ascites		
1	60	66.7
2	12	13.3
3	12	13.3
4	6	6.7
BCLC staging		
A	12	13.3
B	42	46.7
D	12	13.3
C	24	26.7
No prognoses (A + B)	54	60.0
Prognoses (D + C)	36	40.0
Child score		
Mean ± SD	6.46 ± 1.36	
Median (IQR)	6.0 (6.0–7.0)	
MELD score		
Mean ± SD	8.40 ± 7.89	
Median (IQR)	6.50 (2.0–12.0)	

### Differential Expression of Serum miRNAs in Non-Malignant HCV and HCV-Related HCC Patients

**FIGURE 1:**
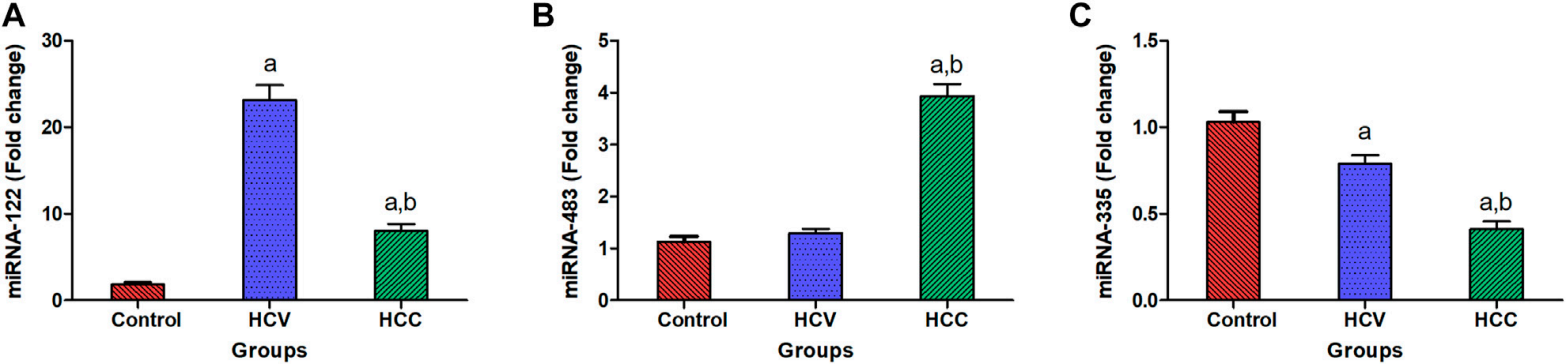
Expression levels of miRNA-122, miRNA-483, and miRNA-335 in serum. **(A)** Serum expression of miRNA-122 in patients with HCC (n = 90) and HCV (*n* = 90) as compared to healthy people (*n* = 60). **(B)** Relative to healthy people (*n* = 60), the fold change in serum expression miRNA-483 in patients with HCC (*n* = 90) and HCV (*n* = 90). **(C)** Fold change in the serum expression miRNA-335 level in HCC (*n* = 90) and HCV (*n* = 90) patients compared to healthy persons (*n* = 60). Values are expressed as mean ± SEM (95% CI). **(A)** Significant difference from the control group at *p* < 0.05. **(B)** Significant difference from the HCV group at *p* < 0.05.

In HCC patients, serum miR-122 was upregulated significantly with a mean fold change of 8.0 ± 0.81 (*p* < 0.005) in comparison with healthy subjects. However, it was markedly overexpressed in the HCV group with 23.17 ± 1.69 fold change (*p* < 0.005) compared to the healthy subjects (Figure 1A). Additionally, HCC subjects showed a significant upregulation in serum miR-483 compared to the healthy controls and the HCV groups with a mean fold change of 3.933 ± 0.228 from the healthy control group (*p* < 0.005) (Figure 1B). However, in HCC patients, serum miR-335 was downregulated significantly with a mean fold change of 0.412 ± 0.0442 (*p* < 0.005) in comparison with healthy subjects. Moreover, serum miR-335 levels in the HCC group were significantly lower than those in the HCV group (0.789 ± 0.049 fold change) (*p* < 0.005) (Figure 1C).

### Diagnostic Performance of Serum miRNA-122, miRNA-483, and miRNA-335

**FIGURE 2:**
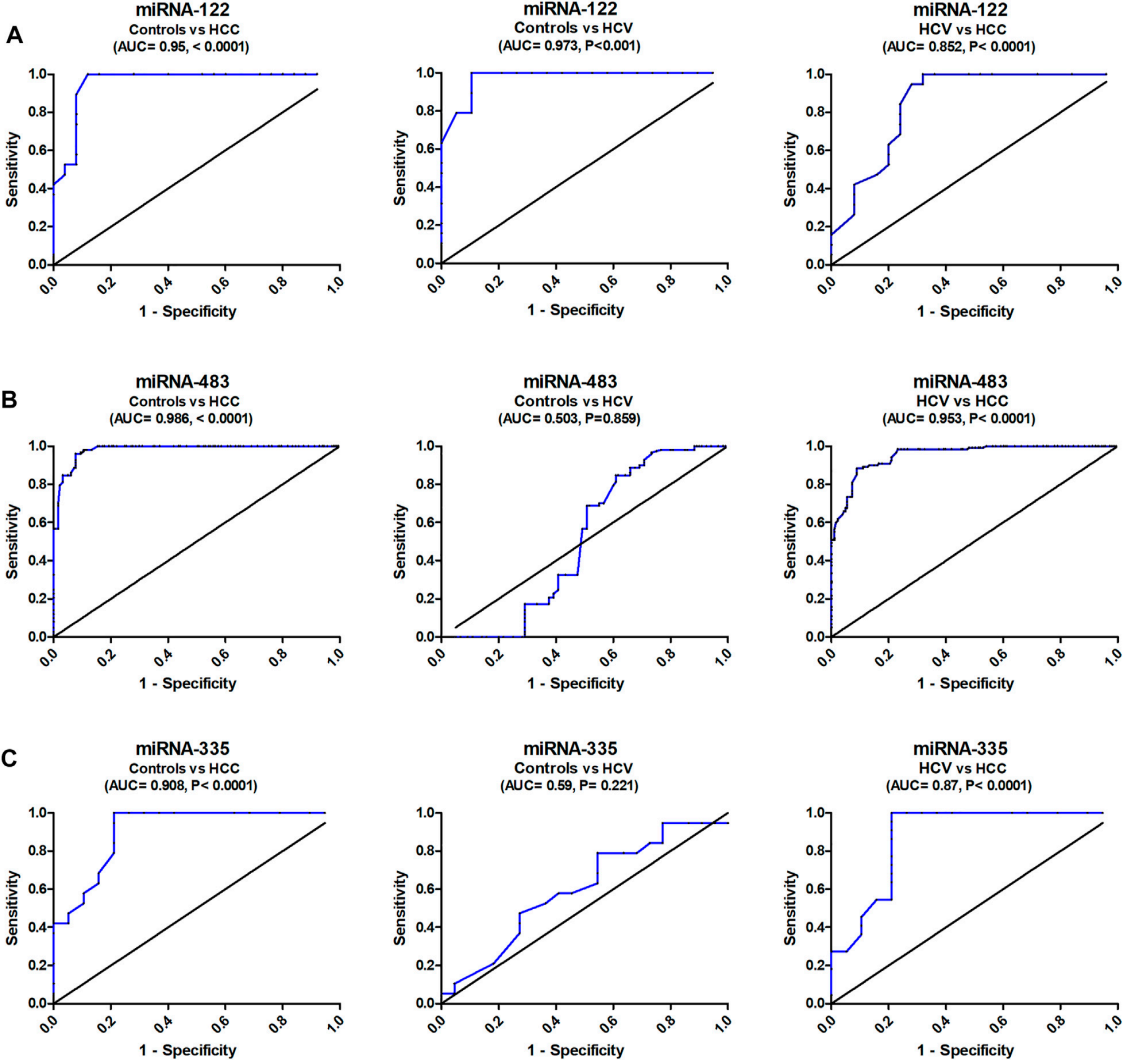
Diagnostic accuracy of serum miRNA-122, miRNA-483, and miRNA-335. Study of the ROC curve of serum miRNA-122, miRNA-483, and miRNA-335 to differentiate the studied types, HCC (*n* = 90) and HCV (*n* = 90), from controls (*n* = 60).

With an AUC of 0.95, serum miR-122 was able to distinguish healthy controls from HCC in ROC curve analysis, with CI = 0.8977 to 1.016, *p* = 0.0001, with sensitivity = 100 percent, specificity = 84.1 percent, at a cutoff <6.55-fold. Serum miR-122 discriminated healthy controls from HCV patients with AUC = 0.97, 95% CI = 0.9313 to 1.016, *p* < 0.0001, with sensitivity = 100%, specificity = 89.5%, at a cutoff <12.5-fold. Also, serum miR-122 discriminated HCV from HCC with AUC = 0.85, 95% CI = 0.7389 to 0.9664, *p* < 0.0001, with sensitivity = 100%, specificity = 71%, at a cutoff >10.6-fold (Figure 2A).

The analysis of ROC curve also revealed that serum miR-483 discriminated healthy controls from HCC with AUC = 0.986, 95% CI = 0.917–1.012, *p* < 0.0001, with sensitivity = 100%, specificity = 82.3%, at a cutoff <2.43-fold. Serum miR-483 also distinguished controls from HCV patients with AUC = 0.503, 95% CI = 0.4285 to 0.5841, *p* = 0.859, with sensitivity = 70%, specificity = 43.2%, at a cutoff <1.205-fold. In addition, it distinguished HCV from HCC with AUC = 0.953, 95% CI = 0.927–1.01, *p* < 0.0001, with sensitivity = 88%, specificity = 91% (at a cutoff <1.98-fold) (Figure 2B).

ROC curve analysis correspondingly showed that serum miRNA-335 discriminated healthy controls from HCC with AUC = 0.908, 95% CI = 0.824 to 1.006, *p* < 0.0001, with sensitivity = 100%, specificity = 79.8%, at a cutoff >0.49-fold**.** It also distinguished controls from HCV with AUC = 0.59, 95% CI = 0.414 to 0.776, *p* = 0.221, with sensitivity = 79%, specificity = 36.3%, at a cutoff >0.73-fold (Figure 2C). Serum miRNA-335 also distinguished HCV from HCC patients with AUC = 0.875, 95% CI = 0.7570 to 0.9942, *p* < 0.001, with sensitivity = 95.4%, specificity = 78.1%, at a cutoff >0.61-fold.

In addition to ROC curve analysis, univariate logistic regression was conducted to select the predictor variables associated with HCC risk among non-malignant groups (Table 4). Serum miR-483 and miR-335 were selected as significant predictor variables in the univariate analysis, with adjustment for age and sex.

**TABLE 4:** Logistic regression analysis to predict the risk of *HCC in non*-malignant groups.

Parameter	Coefficient	SE	*p* ^a^ value	Odds ratio	95% confidence interval
Univariate analysis					
miR-335	0.138	0.028	**< 0.001**	1.345	1.05–1.935
miR-483	0.748	0.184	**< 0.001**	2.3	1.283–3.421

^a^Adjusted for age and sex. HCC, *n* = 90; non-malignant (healthy controls + HCV), *n* = 150. *p* values in bold are statistically significant as *p* < 0.05.

## Discussion

Cancer detection now has a low sensitivity since many cancers are not detected early enough, delaying therapy until it is too late (Fiala and Diamandis, 2018). MiRNA expression is typically dysregulated in cancer (Shaker et al., 2020), resulting in a distinct expression profile that aids in early cancer detection. MiRNAs linked to tumor growth are overexpressed, while suppressors are under-expressed (Shaker et al., 2021). As a result, tissue-specific miRNAs are becoming more widely used in cancer diagnosis (Drees and Pegtel, 2020; Zaafan and Abdelhamid, 2021). The focus of this research was to investigate the expression profiles of selected miR-122, miR-483, and miR-335 in sera from Egyptian patients with HCV-related HCC in order to be used as new non-invasive markers for HCV-related HCC diagnosis.

The expression of miRNA-122 is constrained to the liver, where it is assumed to account for 70 percent of all miRNA expression (Fu and Calin, 2018). In the present research, serum expression levels of miR-122 in the HCV and HCC groups were significantly higher than those in the normal control group (*p* value 0.01), although serum expression levels of miR-122 in the HCC group were significantly lower than those in the HCV group (*p* value 0.01). According to recent research, miRNA-122 expression levels decrease during hepatocarcinogenesis, suggesting that miRNA-122a can act as a tumor suppressor. Our findings were in line with earlier research, which found a decrease in miRNA-122 levels in HCC patients compared to HCV patients (Luo et al., 2013). The levels of miRNA-122, on the contrary, have arisen significantly in the HCV group, implying that miRNA-122 levels increase significantly during hepatocyte damage and subsequently fall dramatically once the liver enters carcinogenesis. According to a study by Jiang *et al.* [25], miRNA-122a levels in the blood have been found to be higher in HCC patients than in healthy people. Matching with our results (El- Garem et al., 2014), Trebicka et al. (2013) examined hepatic miR-122 expression in HCV-linked HCC in comparison with healthy liver samples and found that miR-122 was significantly upregulated in malignant liver nodules compared to healthy liver. They hypothesized that miR-122 would suppress the expression of undiscovered tumor suppressor genes, resulting in tumor development (Trebicka et al., 2013). miR-122 expression was considerably higher in ccRCC tissues with lymphatic invasion than those without lymphatic invasion, despite the fact that it was unrelated to clinical stages and grades. While more research on the exact process is required, the findings of this study are congruent with those of Lian et al., who found that miR-122 expression increased cancer cell malignancy characteristics in RCC cells (Lian et al., 2013), reliable with the preceding study in ccRCC.

Previous research has demonstrated higher expression of miR-483 in HCC patients’ serum (Gui et al., 2011; Shen et al., 2013), which is compatible with our current findings, which show considerable elevation of miRNA-483 in HCV-related HCC patients compared to HCV and healthy control groups. miR-483 was revealed to function as an anti-apoptotic oncogene with a pathogenic effect and molecular method of action in cancer cell lines (HEPG2, liver carcinoma, and HCT116, colorectal carcinoma) (Taheri et al., 2021). An *in vitro* investigation of adrenocortical cancer indicated that miR-483-5p promotes proliferation. These findings show that miR-483 may play a carcinogenic role in carcinogenesis (Yang et al., 2017). Several earlier investigations in Wilms tumor, colorectal cancer, malignant pheochromocytoma, and hepatocellular carcinoma tissues demonstrated positive relationships between miR-483 expression and IGF-II mRNA levels, supporting this theory (Ma et al., 2011). miR-483-5 expression was also observed to be higher in serum from hepatocellular cancer patients in a previous investigation (Shen et al., 2013), which is consistent with our existing observation.

In the current investigation, we discovered that HCV-related HCC patients had significantly lower levels of miRNA-335 expression than HCV patients and healthy controls. miR-335 has been demonstrated to behave as a tumor suppressor and inducer in a variety of cancer types in numerous investigations (Slattery et al., 2015; Wang et al., 2017). Many cancers, including breast cancer, lung cancer, colorectal cancer, and ovarian cancer (Heyn et al., 2011), have dysregulated miR-335, which serves as an oncogene or tumor suppressor in many malignant tumors. Furthermore, miR-335’s impact on the biological effect of numerous malignancies, including proliferation, apoptosis, migration, and invasion, is critical to its function in cancer (Liang et al., 2019). miR-335 has been shown to impact proliferation and apoptosis in a number of malignancies in several research studies. The serine/threonine kinase protein Rho-associated coiled-coil containing protein kinase 1 (ROCK1) promotes the growth of malignant tumors (Cui et al., 2015). miR-335 inhibits cell proliferation and cell cycle progression in NSCLC by suppressing ROCK1 expression. In HCC, miR-335 can influence cell proliferation, migration, and invasion in addition to cell proliferation. miR-335 inhibits ROCK1 expression by binding directly to its untranslated region, resulting in decreased cell proliferation and migration in cancer development (Ye et al., 2021). According to our results, the downregulation of miR-335 observed in HCC group patients increases the ROCK1 expression which facilitates the development and the progression of HCC.

The ROC curve was constructed to test the diagnostic ability of miRNA-122, producing a diagnostic accuracy (AUC = 95%) and a sensitivity of 100% in distinguishing HCC cases from controls. MicroRNA-483 had a high diagnostic accuracy (AUC = 98.6%) and 100% sensitivity for distinguishing healthy controls from HCC patients. It had a respectable capability to discriminate between HCV and HCC (AUC = 95.3%) and an 88 percent sensitivity. For distinguishing control subjects from HCC patients, microRNA-335 demonstrated a good diagnostic accuracy (AUC = 90.8 percent) and 100 percent sensitivity. It established a notable ability to differentiate HCV from HCC (AUC = 87.5% and sensitivity of 95.4%).

To summarize, early identification of HCV-related HCC is crucial for efficient disease therapy and can improve the survival rate of HCC patients. The findings of the current study highlighted the possible use of miR-122, miR-483, and miR-335 as potential diagnostic and prognostic biomarkers for clinical application. The current study elucidates that serum miRNA-335 expression is significantly under-expressed in patients with HCV-related HCC, while the expressions of miR-122 and miRNA-483 are overexpressed compared to those in the non-malignant HCV patients. The miRNA-122, miR-483, and miR-335 panel presented a great diagnostic potential as it demonstrated the highest diagnostic accuracy for discriminating non-malignant HCV patients from HCV-related HCC patients.

## Data Availability

The datasets presented in this study can be found in online repositories. The names of the repository/repositories and accession number(s) can be found in the article/Supplementary Material.
